# Common Chemical Plasticizer Di(2-Ethhylhexyl) Phthalate Exposure Exacerbates Coxsackievirus B3 Infection

**DOI:** 10.3390/v16121821

**Published:** 2024-11-23

**Authors:** Ramina Kordbacheh, Madelyn Ashley, William D. Cutts, Taryn E. Keyzer, Shruti Chatterjee, Tyler J. Altman, Natalie G. Alexander, Timothy E. Sparer, Brandon J. Kim, Jon Sin

**Affiliations:** 1 Department of Biological Sciences, University of Alabama, Tuscaloosa, AL 35487, USA; rkordbacheh@crimson.ua.edu (R.K.); mbashley1@crimson.ua.edu (M.A.); wdcutts@crimson.ua.edu (W.D.C.); tekeyzer@crimson.ua.edu (T.E.K.); schatterjee6@ua.edu (S.C.); tjaltman1@crimson.ua.edu (T.J.A.); nalexander6@crimson.ua.edu (N.G.A.); bjkim4@ua.edu (B.J.K.); 2 Department of Microbiology, University of Tennessee, Knoxville, TN 37996, USA; tsparer@utk.edu

**Keywords:** coxsackievirus B3 (CVB), Di(2-ethhylhexyl) phthalate (DEHP), proviral, environmental factors

## Abstract

Di(2-ethhylhexyl) phthalate (DEHP) is a common plastic rubberizer. DEHP leaches from plastic matrices and is under increasing scrutiny as numerous studies have linked it to negative human health manifestations. Coxsackievirus B3 (CVB) is a human pathogen that typically causes subclinical infections but can sometimes cause severe diseases such as pancreatitis, myocarditis, and meningoencephalitis. Though CVB infections are common, severe illness is relatively rare, and it is unclear what factors mediate disease severity. In this study, we sought to determine the effects that DEHP has on CVB infection in a variety of human cell types to evaluate whether this plastic-derived pollutant could represent a proviral environmental factor. Methods: HeLa cervical cancer cells, human induced pluripotent stem cell-derived brain-like endothelial cells (iBECs), and Caco-2 colon carcinoma cells were exposed to 40 µg/mL DEHP for 24 h prior to infecting with enhanced green fluorescent protein (EGFP)-expressing CVB. The severity of the infection was evaluated via fluorescence microscopy and flow cytometry-based viral EGFP detection, viral plaque assay on tissue culture media, and Western blotting to detect VP1 viral capsid protein. Interferon-associated proteins such as interferon regulatory factor (IRF) 3, IRF7, interferon-induced transmembrane (IFITM) 2, and IFITM3 were measured by Western blotting. The roles of IFITM2 and IFITM3 in the context of CVB infection were evaluated via siRNA silencing. Results: We found that DEHP drastically increased CVB infection in each of the cell types we tested, and, while the cellular processes underlying DEHP’s proviral properties were not entirely clear, we observed that DEHP may subvert CVB-induced interferon signaling and elevate levels of IFITMs, which appeared to bolster CVB infection. Conclusions: DEHP may represent a major environmental factor associated with the severity of CVB infection. Further understanding of how DEHP exacerbates infection may better elucidate its potential role as a proviral environmental factor.

## 1. Introduction

Di(2-ethylhexyl) phthalate (DEHP) is one of the most common plasticizers applied to polyvinyl chloride (PVC) to rubberize this typically hard plastic and make it more pliable [1,2]. DEHP is regularly used in a number of household items, and food packaging and medical products like blood bags, tubing, disposable gloves, and oxygen masks [3,4,5,6,7]. As DEHP is not strongly bound to PVC matrices, it can readily leach out of plastics and can increase the longevity of stored blood [8,9]. This is one of the primary reasons that DEHP is still a standard component of blood bags, despite ongoing efforts to identify alternative plasticizers. Because DEHP accumulates both in the environment and within bodily tissues, and there are links to human health abnormalities such as liver cancer and cardiovascular diseases, and in the reproductive system, there is a push to transition away from its usage [8,10,11]. Studies on the association between DEHP and infectious diseases are currently very limited. However, patients exposed to increased concentrations of DEHP have exacerbated dengue virus infections and associated clinical symptoms [12].

To investigate this possible connection between DEHP and viral infections, our initial step was to use coxsackievirus B3 (CVB) as a model RNA virus. CVB is a positive sense single-stranded RNA virus and a member of the *Enterovirus* genus and *Picornavaridae* family [13,14,15]. Like other picornaviruses, CVB is a naked virus lacking a lipid envelope [16]. Human exposure to CVB is relatively common worldwide and is typically either asymptomatic or leads to mild subclinical illness, including rash, fever, or upper-respiratory illness. Occasionally though, CVB infections can be much more severe, resulting in potentially fatal systemic diseases such as myocarditis, pancreatitis, and meningoencephalitis [17,18]. All ages are susceptible to CVB infection and disease; however, the most affected group is typically children under 2 years of age [19]. Though it is thought that a large proportion of the population has at some point been infected with CVB, cases of severe disease are comparatively low. Generally, CVB disease manifestation may be influenced by various genetic and environmental factors [20,21]. Despite recognizing the influence of environmental factors, the specific factors that may impact the severity of CVB infection have not been identified.

In this study, we examined the effect of DEHP exposure on CVB infection in three different human cell types: cervical cancer cells (HeLa), induced pluripotent stem cell-derived brain-like endothelial cells (iBEC), and colon carcinoma cells (Caco-2). We additionally tested the effects on mouse atrial cardiomyocytes (HL-1). We found that media concentrations of 40 µg/mL DEHP had no observable impact on cell growth or viability in any of the cell types. However, this dose of DEHP substantially enhanced CVB infection compared with vehicle-treated controls. Given the disparity in the cell types used, this suggests that the proviral effects that DEHP has on CVB infection is not particularly tissue-specific. In several of these cell types, increased infection appears to be a byproduct of dramatically enhanced viral spread, as infected cell number and extracellular viral titers were disproportionately higher than intracellular viral protein content. Because CVB is a leading cause of meningoencephalitis, we further interrogated cellular factors in iBECs to determine the contributions to DEHP-mediated increases in infection. We found that DEHP significantly blunted interferon regulatory factor 7 (IRF7) in response to viral infection. We further examined interferon-related responses and assessed levels of interferon-induced transmembrane proteins (IFITMs) in HeLas and iBECs exposed to DEHP. Surprisingly, we found that IFITM2/3 was increased with DEHP treatment, and genetically silencing *IFITM2* and *IFITM3* significantly reduced infection. Little is currently known regarding the relationships between CVB and IFITMs; however, our data suggest that IFITM2/3 may support infection. Seeing that this DEHP-mediated elevation in IFITM2/3 occurs while IRF7 is inhibited, this may suggest that IFITM2/3 may be induced independent of type I interferon signaling. To determine whether DEHP’s proviral effects are specific to CVB or viruses in general, we repeated these experiments with murine CMV (MCMV), a large DNA virus, and the bacterium group B streptococcus (GBS). In all, we show that CVB and GBS infection is significantly enhanced following DEHP exposure while MCMV is not. This suggests that, while DEHP might not be indiscriminately proviral, it can bolster some bacterial infections. Further studies are needed to fully understand the underlying mechanisms that contribute to the proviral/bacterial nature of DEHP and to elucidate the physiological impact of DEHP on disease.

## 2. Materials and Methods

### 2.1. Cell Culture

HeLa human cervical cancer cells were cultured in Dulbecco’s Modified Eagle’s Medium (DMEM) (Sigma-Aldrich, St. Louis, MO, USA, D6429) complete medium containing 10% fetal bovine serum (FBS) (Avantor, Radnor, PA, USA, 89510-166) and 100 µg/mL penicillin/streptomycin (Sigma-Aldrich, St. Louis, MO, USA, A5955). Human-induced pluripotent stem cell (iPSC)-derived brain-like endothelial cells (iBECs) were cultured in human endothelial cell serum-free medium containing 1% B27 (Gibco, Grand Island, NY, USA, 17504044). Colorectal cancer cells (Caco-2s) were cultured in DMEM containing 10% FBS. Mouse atrial cardiomyocyte cells (HL-1s) were cultured in Claycomb medium (Sigma-Aldrich, St. Louis, MO, USA, 51800C) containing 10% FBS, 100 µg/mL penicillin/streptomycin, 0.1 mM norepinephrine (Thermo Fisher Scientific, Waltham, MA, USA, L08087.03), and 2 mM L-glutamine (Gibco, Grand Island, NY, USA, 25030-081). The 10.1 mouse embryonic fibroblasts (Fiorenza Ianzini, University of Iowa, Iowa City, IA, USA) [22] were cultured in DMEM containing 10% FBS and 100 µg/mL penicillin/streptomycin. All the cell types were cultured at 37 °C in 5% CO_2_.

### 2.2. Differentiation of Induced Pluripotent Stem Cells into Brain-like Endothelial Cells

iPSCs (WiCell Research, Madison, WI, USA, ID: WISCi004-B) were cultured on Matrigel-coated plasticware, maintained in StemFlex medium (Gibco, Grand Island, NY, USA, A3349401), and routinely passaged. Once iPSCs reached 80% confluency, cells were differentiated according to previously published protocols [23,24,25]. Cells were maintained in a StemFlex medium for 3 days. To initiate differentiation into brain-like endothelial cells, cells were then cultured in an unconditioned medium for 6 days. Lastly, cell media were changed to an endothelial cell medium supplemented with 10 µM retinoic acid (Sigma-Aldrich, St. Louis, MO, USA, R2625) to enhance blood–brain barrier phenotypes such as high TEER [25]. Purified iBECs were maintained in an endothelial cell medium containing 1% B27 (Gibco, Grand Island, NY, USA, 17504044) on extracellular matrix-coated plasticware.

### 2.3. Generation of Enhanced Green Fluorescent Protein-Expressing Coxsackievirus B3 Viral Stocks

Viral stocks were established using the previously described enhanced green fluorescent protein (EGFP)-containing pMKS1 plasmid (Ralph Feuer, San Diego State University, San Diego, CA, USA) [26]. This plasmid not only contains the entire backbone of the myocarditis Nancy H3 variant of CVB3, but also has a unique SfiI restriction site upstream of the viral backbone wherein EGFP was inserted. Viral transcripts were generated by linearizing the plasmid by digesting with ClaI restriction enzyme and performing in vitro transcription on digested products via the mMessage mMachine T7 transcription kit (Thermo Fisher Scientific, Waltham, MA, USA,) following the manufacturer’s protocol. Transcription products were then transfected into HeLa cells using Lipofectamine 2000 (Thermo Fisher Scientific, Waltham, MA, USA, 11668027) following the manufacturer’s protocol. Once 50% of cells displayed viral EGFP, cells were scraped, media and cells were collected and subjected to three freeze–thaw cycles, and cellular debris was removed by centrifuging at 800× *g*. Supernatants were referred to as “passage 1”. Viral stocks were expanded by overlaying the “passage 1” virus onto fresh HeLa cells, which were similarly harvested, freeze-thawed, and clarified once 50% of cells displayed viral EGFP. The resulting supernatant was deemed “passage 2” and used for downstream infection experiments. The HeLa cell growth medium was used for mock infections.

### 2.4. DEHP Treatment

Di(2-ethylhexyl) phthalate (DEHP) (Sigma-Aldrich, St. Louis, MO, USA, 80030-5ML) was diluted in dimethyl sulfoxide (DMSO) (Sigma-Aldrich, St. Louis, MO, USA, D8418) at a concentration of 40 mg/mL. HeLas, iBECs, Caco-2s, HL-1s, and 10.1 MEFs were treated with 40 µg/mL DEHP by diluting DEHP stock 1:1000 in a respective cell medium for 24 h prior to infecting with EGFP-CVB at indicated multiplicities of infection for indicated amounts of time. Vehicle controls were treated with an equivalent volume of DMSO. A 40 µg/mL DEHP concentration is considered within a clinically relevant range [27,28]. Though no cell death was observed at any concentration tested, 80 µg/mL resulted in a reduced cell number after treatment.

### 2.5. Western Blot

Cells were harvested in a radioimmunoprecipitation assay (RIPA) buffer (50 mM Tris-HCl (Sigma-Aldrich, St. Louis, MO, USA, 10812846001), 1% NP-40 (Sigma-Aldrich, St. Louis, MO, USA, 74385), 0.5% deoxycholate, 0.1% sodium dodecyl sulfate, 150 mM sodium chloride (Sigma-Aldrich, St. Louis, MO, USA, 13423), 2 mM ethylenediaminetetraacetic acid), and a protease inhibitor cocktail (Thermo Fisher Scientific, Waltham, MA, USA, A32965). Protein concentrations were determined using bicinchoninic acid assay (Thermo Fisher Scientific, Waltham, MA, USA, 23228). Equal amounts of protein were prepared in a Laemmli sample buffer (1% bromophenol blue (Allied Chemical, Morris Township, NJ, USA 0332), 1.5 M Tris-Cl pH 6.8, glycerol (Sigma-Aldrich, St. Louis, MO, USA, G5516), β-mercaptoethanol (Fisher Scientific, Hampton, NH, USA, BP176-100) and then separated in 4–12% Bis-Tris protein gels. Proteins were then transferred to nitrocellulose membranes, and membranes were stained with Ponceau S solution (Sigma-Aldrich, St. Louis, MO, USA, SLCQ5486) and imaged via the iBright FL1500 Imaging System (Invitrogen, Waltham, MA, USA). Membranes were blocked in a blocking solution (Tris-buffered saline containing 0.1% Tween-20 (Sigma-Aldrich, St. Louis, MO, USA, P1379) (TBS-T) and 3% bovine serum albumin (BSA) (Sigma-Aldrich, St. Louis, MO, USA, A9647), and placed in a primary antibody diluted in the blocking solution and incubated overnight at 4 °C. Primary antibodies used in this study were as follows: VP1 (Mediagnost, Reutlingen, Germany, Cox mAB 31A2, 1:2000), IRF7 (Santa Cruz Biotechnology, Dallas, TX, USA, sc-74472, 1:1000), IRF3 (Santa Cruz Biotechnology, Dallas, TX, USA, sc-33641, 1:1000), and IFITM2/3 (Cell Signaling Technology, Danvers, MA, USA, E5F8C, 1:1000). Membranes were then washed in TBS-T and incubated in an anti-mouse secondary antibody (Sigma-Aldrich, St. Louis, MO, USA, 12-349, 1:3000) and anti-rabbit secondary antibody (Sigma-Aldrich, St. Louis, MO, USA, F9887, 1:3000). Membranes were again washed in TBS-T, and, after applying SuperSignal West Dura Extended Duration chemiluminescent substrate, were imaged via the iBright FL1500 Imaging System. Densitometry was performed using ImageJ software (National Institutes of Health, Bethesda, MA, USA, version 1.53), with background subtraction applied to all quantifications.

### 2.6. Plaque Assay

Infectious tissue culture media were serially diluted in DMEM and 400 µL of each dilution was overlaid onto confluent HeLa cell monolayers in 6 well plates. Following one hour incubation at 37 °C with occasional rocking, cells were overlaid with 4 mL 50:50 mixture of 1.2% molten agarose (VWR, 1855C495) and 2X DMEM and incubated at 37 °C. After 48 h, agar plugs were fixed for 20 min with 2 mL plaque fixative (25% acetic acid (Thermo Fisher Scientific, Waltham, MA, USA, A38S-212) and 75% methanol (Sigma-Aldrich, St. Louis, MO, USA, 179337). Agar plugs were then removed, and cells were stained with a 2.34% crystal violet solution (Sigma-Aldrich, St. Louis, MO, USA, C0775). The crystal violet was then washed off and viral plaques were counted.

MCMV plaque assays were carried out as previously described [29]. Briefly, MEF 10.1 cells were used to determine viral titers. Cells were plated in a six-well dish. Supernatants from treated or non-treated cells were serially diluted and added to MEF 10.1 cells and incubated for one hour. After incubation, the diluted virus was removed, and cells were overlaid with a carboxymethyl cellulose (CMC) (Sigma-Aldrich, St. Louis, MO, USA, 419273) medium and incubated for 3–5 days. CMC was removed, and plates were stained with Coomassie blue (Sigma-Aldrich, St. Louis, MO, USA, G1041), and plaques were counted.

### 2.7. Cell Viability

Supernatants from cultured cells were first collected in individual 15 mL conical tubes. Cells were then washed with phosphate-buffered saline (PBS) (Thermo Fisher Scientific, Waltham, MA, USA, J60801.K7) and the wash was added to respective conical tubes to ensure the collection of already detached cells. Adherent cells were detached with trypsin-EDTA (Thermo Fisher Scientific, Waltham, MA, USA, 25200056) and collected in respective conical tubes. Cells were pelleted by centrifugation at 300× *g* for 5 min. After resuspending cells in 1 mL of fresh growth medium, equal volumes of suspended cells and trypan blue solution (Thermo Fisher Scientific, Waltham, MA, USA, T10282) were combined, and viability was quantified using a Countess 3 cell counter (Thermo Fisher Scientific, Waltham, MA, USA).

### 2.8. Flow Cytometry

Supernatants from cultured cells were first collected in individual 15 mL conical tubes. Cells were then washed with PBS and the wash was added to respective conical tubes to ensure the collection of already detached cells. Adherent cells were detached with trypsin-EDTA and collected in respective conical tubes. A 10 mL growth medium was added to each conical tube and centrifuged at 500× *g* for 10 min. Supernatant was discarded and cells were resuspended and fixed in 5 mL 4% paraformaldehyde (PFA) (Sigma-Aldrich, St. Louis, MO, USA, 47608-1L-F). After 10 min fixation, cells were centrifuged at 500× *g* for 10 min. The supernatant was discarded and cells were washed by resuspending in 10 mL PBS and centrifuging at 500× *g* for 10 min. The supernatant was discarded and cells were resuspended in 500 µL fresh PBS. Cells were then filtered in filter tubes to ensure single cell suspension and analyzed via an Attune NxT flow cytometer (Thermo Fisher Scientific, Waltham, MA, USA).

### 2.9. Transendothelial Electrical Resistance

Transendothelial electrical resistance (TEER) was carried out as previously described [23,24,25]. Briefly, iBECs were seeded on 6.5 mm transwell inserts in a 24-well plate. A total of 24 h before infection, cells were treated with DEHP or an equal concentration of DMSO. Transwells were then infected at MOI 1 with EGFP-CVB or were mock-infected. TEER was measured every 24 h for 9 days using a EVOM^2^ epithelial voltohmmeter (World Precision Instruments, Sarasota, FL, USA)and a chopsticks electrode set STX2 (World Precision Instruments, Sarasota, FL, USA).

### 2.10. Bacterial Adherence and Infection Assays

Adherence and invasion were quantified as described previously [30,31,32]. iBECs were seeded onto collagen (Sigma-Aldrich, St. Louis, MO, USA, C5533)/fibronectin (Sigma-Aldrich, St. Louis, MO, USA, F1141)-coated 24-well plates (Corning, Corning, NY, USA, CLS3527) and grown to a confluent monolayer. A total of 24 h prior to infection, cells were treated with either DEHP or an equal concentration of DMSO. Overnight cultures of *S. agalactiae* were grown in Todd Hewitt broth media. iBECs were then infected with *S. agalactiae* at an MOI of 10. During infection, cells were incubated at 37 °C + 5% CO_2_. For adherence, cells were infected for 30 min, then washed five times with sterile PBS to remove non-adherent bacteria. Following the washes, the wells were treated for 10 min with 0.25% trypsin-EDTA (VWR), and the mammalian cells were lysed with 0.025% Triton X-10 (Sigma-Aldrich, St. Louis, MO, USA, X100). The resultant suspensions were diluted and plated onto Todd Hewitt agar (THA) plates. For invasion, cells were infected as above for 2 h, followed by the addition of 100 µg/mL gentamycin and incubation for an additional 2 h at 37 °C + 5% CO_2_. Cells were then washed 3x in sterile PBS and then lysed as above before being plated onto THA plates. Relative adherence and invasion were quantified with the following formula: (#CFU * dilution correction * volume correction)/(input CFU/well).

### 2.11. Small Interfering RNA (siRNA) Transfection

Scrambled siRNA (Santa Cruz Biotechnology, Dallas, TX, USA, sc-37007), human *IFITM2* siRNA (Santa Cruz Biotechnology, Dallas, TX, USA, sc-96760), and human *IFITM3* siRNA (Santa Cruz Biotechnology, Dallas, TX, USA, sc-97053) were reconstituted in nuclease-free water following the manufacturer-provided datasheet. siRNAs were transfected into HeLa cells using an Effectene Transfection Reagent (Qiagen, Valencia, CA, USA, 301425) according to the manufacturer’s guidelines for reagent volumes. After 48 h of transfection, HeLa cell media was refreshed with a DMEM complete medium. Cells were then infected with EGFP-CVB at MOI 0.001.

### 2.12. Quantification and Statistical Analysis

GraphPad Prism version 10.2.1 was used for all statistics calculations. For pair-wise comparisons, Student’s t-tests were performed, and for multiple comparisons, one-way ANOVA was performed. GraphPad Prism area under the curve calculation was utilized to determine differences in barrier integrity representing the entire time course. Statistical significance was accepted if *p* < 0.05.

## 3. Results

### 3.1. Coxsackievirus B3 Infection Increases Following Di(2-Ethhylhexyl) Phthalate (DEHP) Exposure

**Figure 1:**
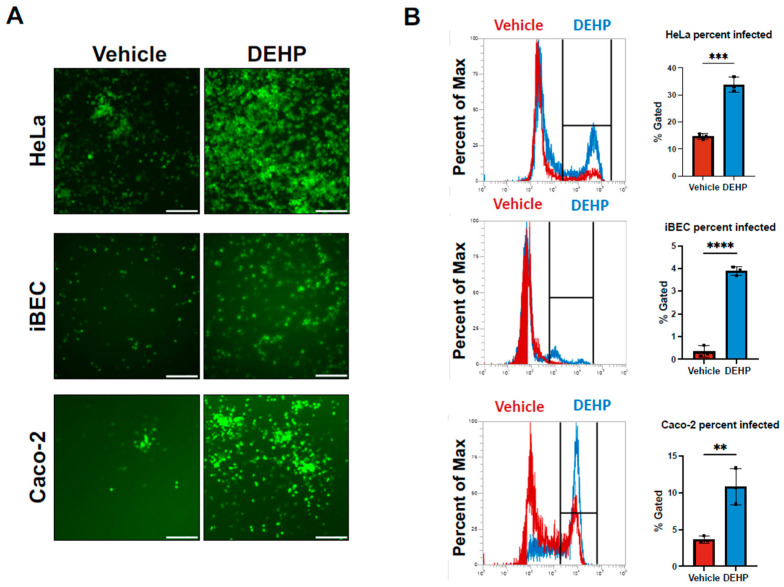
CVB infection increases following DEHP exposure. (**A**) Fluorescence microscopy detecting enhanced green fluorescent protein (EGFP) expression between HeLa cells (top), iBECs (middle), and Caco-2 cells (bottom) treated with 40 µg/mL DEHP or equivalent volume vehicle for 24 h prior to infecting with EGFP-CVB at multiplicity of infection (MOI) of 0.001, 1, or 0.001, respectively, for 24 h or 48 h (scale bars = 100 µm). (**B**) Flow cytometry quantifying cells in (**A**). Flow cytometry gates for quantification were defined using the termination of the fluorescent peak, at which the fluorescent signal was closest to zero, in a mock uninfected sample. This allowed for effective measurement of a shift in intensity or appearance of an infected population. (Student’s t-test; n = 3 per group; ** *p* < 0.01, *** *p* < 0.001, **** *p* < 0.0001. Error bars represent standard deviation.)

To begin interrogating the effects of Di(2-ethhylhexyl) phthalate (DEHP) exposure on coxsackievirus B3 (CVB) infection, we treated three different human cell types with DEHP and infected them with enhanced green fluorescent protein-expressing CVB (EGFP-CVB). HeLa cells, iBECs, and Caco-2 cells were treated with 40 µg/mL DEHP for 24 h and subsequently infected with EGFP-CVB. This concentration of DEHP is considered within a clinically relevant range [27,28], and treatment with DEHP alone had no observable toxic effect on cell viability, proliferation, or morphology (Supplemental Figure S1). However, fluorescence microscopy on infected cells revealed substantially increased numbers of EGFP-positive cells in infected DEHP-treated groups compared with their corresponding infected DMSO-treated (vehicle) groups (Figure 1A). We also examined CVB infection following DEHP exposure on HL-1 cells and observed increased infected cell numbers 24 h post-infection in the DEHP-treated group (Supplemental Figure S2A). To quantitatively validate these microscope-based observations, we performed flow cytometry on infected cells and indeed found that DEHP treatment significantly increased EGFP positivity (Figure 1B). These results indicate that DEHP significantly enhances infected cell numbers following CVB exposure.

### 3.2. DEHP Enhances Viral Egress of CVB in Certain Cell Types

**Figure 2:**
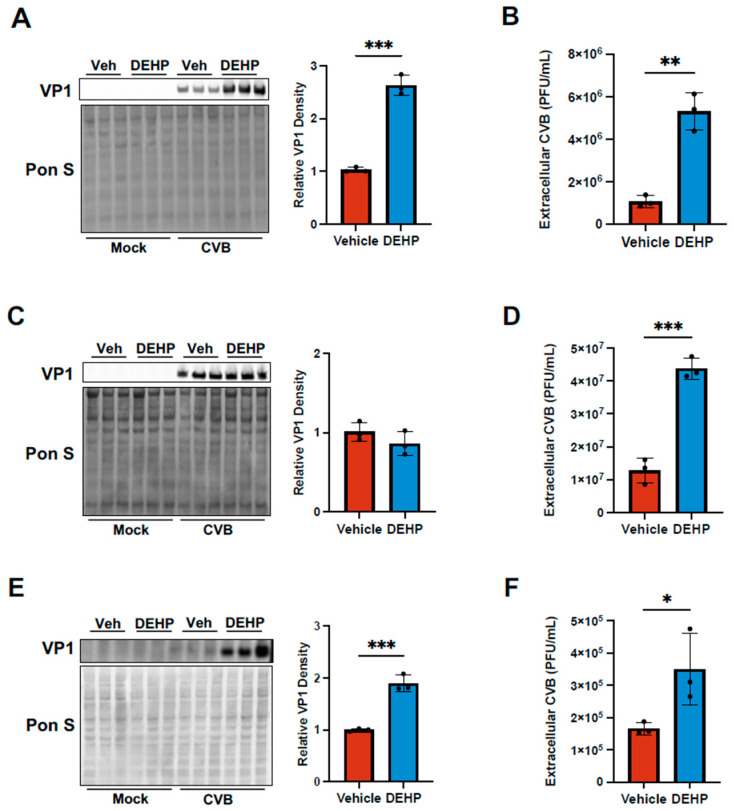
DEHP enhances viral egress of CVB. (**A**) Western blot detecting viral capsid protein VP1 in HeLa cells treated with DMSO (vehicle) or DEHP before infecting with EGFP-CVB or mock-infecting. Ponceau S (Pon S) stain is shown below. The densitometric quantification of VP1 is shown to the right. (**B**) Viral plaque assays enumerating infectious viral titers in tissue culture media from cells in (**A**). (**C**) Western blot detecting VP1 in iBECs treated with vehicle or DEHP prior to infecting with EGFP-CVB or mock-infecting. Pon S stain is shown below. Densitometric quantification of VP1 is shown to the right. (**D**) Viral plaque assays enumerating infectious viral titers in tissue culture media from cells in (**C**). (**E**) Western blot detecting VP1 in Caco-2 cells treated with vehicle or DEHP prior to infecting with EGFP-CVB or mock-infecting. Pon S stain is shown below. Densitometric quantification of VP1 is shown to the right. (**F**) Viral plaque assays enumerating infectious viral titers in tissue culture media from cells in (**E**). (Student’s t-test; n = 3 per group; * *p* < 0.05, ** *p* < 0.01, *** *p* < 0.001. Error bars represent standard deviation.)

Next, we assessed whether DEHP altered intracellular and extracellular viral content following infection. Western blots on whole cell lysates revealed marked increases in viral capsid protein VP1 and substantially higher amounts of extracellular virus in HeLa cells (Figure 2A,B). In the case of iBECs, despite the previously observed increase in infected cells, intracellular VP1 content was not significantly altered in DEHP-treated cells (Figure 2C). Interestingly, extracellular viral titers were significantly elevated in iBECs exposed to DEHP (Figure 2D). HL-1s also showed disproportionately higher levels of extracellular viral titers following DEHP treatment (Supplemental Figure S2B,C). Caco-2 cells were the only cell type that did not exhibit an exaggerated elevation in CVB release, though both VP1 and viral titers in the media were still both significantly enhanced with DEHP (Figure 2E,F). Overall, the disparity between extracellular and intracellular viral loads that we observed in HeLas, iBECs, and HL-1s, coupled with the dramatically increased infected cells (Figure 1), suggests that DEHP can greatly enhance viral spread.

### 3.3. DEHP Suppresses CVB-Mediated Interferon Regulatory Factor 7 Induction in iBECs

**Figure 3:**
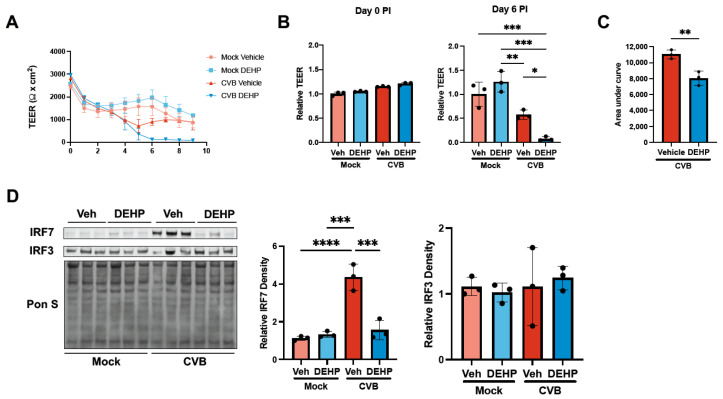
DEHP suppresses CVB-mediated interferon regulatory factor 7 induction in iBECs. (**A**) Transendothelial electrical resistance (TEER) measurements from iBECs treated with vehicle or DEHP prior to infecting with EGFP-CVB or mock-infecting. Daily raw TEER values from day 0 to day 9 post-infection (PI). (**B**) (Left) Relative TEER was recorded immediately after infecting cells (Day 0 PI). Values normalized to day 0 PI mock vehicle group. (Right) Relative TEER recorded day 6 PI. Values normalized to day 6 PI mock vehicle group. (**C**) Area under the curve comparing overall TEER for vehicle CVB and DEHP CVB groups. (**D**) Western blots detecting IRF7 and IRF3 in iBECs treated with vehicle or DEHP prior to infecting with EGFP-CVB or mock-infecting for 48 h. Pon S stain is shown below. Densitometric quantification of IRF7 and IRF3 are shown to the right (Student’s t-test (**C**), ANOVA (**B**,**D**); n = 3 per group; * *p* < 0.05, ** *p* < 0.01, *** *p* < 0.001, **** *p* < 0.0001. Error bars represent standard deviation).

In a previous study assessing DEHP exposure in patients infected with dengue virus, higher urine DEHP concentrations correlated with exacerbated disease symptoms such as chills and gastrointestinal bleeding [12]. Furthermore, this study found that elevated DEHP concentrations correlated negatively with urine concentrations of IL-23 and plasma concentrations of IL-17, both of which are involved in host immunity towards dengue virus. Given that DEHP reportedly limits host antiviral responses, we sought to determine whether the suppression of antiviral immunity may contribute to an enhanced CVB infection following DEHP treatment. Because CVB is a known neurotropic virus, we focused on infection in iBECs, a cell line with robust blood–brain barrier properties. We previously demonstrated a decline in barrier properties following CVB infection as indicated by a dramatic decrease in transendothelial electrical resistance (TEER) [33]. In CVB-infected iBECs, we observed that DEHP caused nearly complete infection-induced loss of barrier integrity by 6 days PI, whereas vehicle-treated infected cells maintained some barrier function (albeit reduced), indicating that DEHP accelerates infection-mediated barrier dysfunction (Figure 3A,B). In addition, area under the curve quantifications measuring cumulative TEER readings from day 0 to day 9 were significantly lower in the CVB DEHP group compared with the CVB vehicle group (Figure 3C). Numerous studies have shown that the brain endothelium possesses robust interferon-mediated antiviral defenses that help restrict passage of viruses into the central nervous system [34,35,36]. We performed Western blots to explore interferon responses in iBECs and found that interferon regulatory factor 7 (IRF7) was significantly elevated following CVB infection alone, however iBECs failed to induce IRF7 in the presence of DEHP (Figure 3D). Interestingly, CVB infection or DEHP treatment of iBECs did not affect interferon regulatory factor 3 (IRF3), another central component of interferon-mediated antiviral signaling. These results suggest that the suppression of antiviral immunity via IRF7 inhibition may contribute to DEHP-mediated proviral effects.

### 3.4. DEHP Exacerbates CVB Infection by Enhancing Interferon-Induced Transmembrane 2 and 3 in HeLas and iBECs

**Figure 4:**
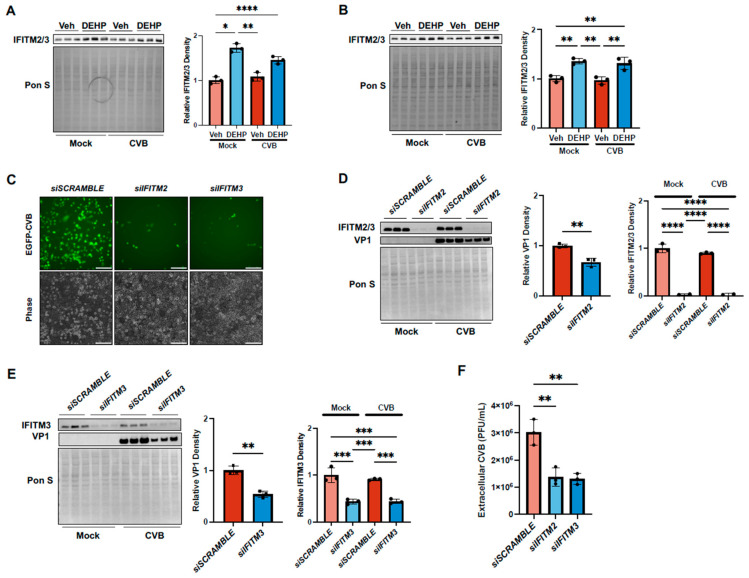
DEHP exacerbates CVB infection by enhancing interferon-induced transmembrane 2 and 3 in HeLas and iBECs. (**A**,**B**) Western blots detecting IFITM2/3 in HeLa (**A**) and iBECs (**B**) treated with vehicle or DEHP prior to infecting with EGFP-CVB or mock-infecting. Pon S stain is shown below. Densitometric quantification of IFITM2/3 is shown to the right. (**C**) Fluorescence microscopy detecting enhanced green fluorescent protein (EGFP) expression between cells transfected with scramble siRNA (*siSCRAMBLE)* (top), *IFITM2* siRNA (*siIFITM2*) (middle), or *IFITM3 siRNA* (*siIFITM3*) (bottom) for 48 h prior to infecting with EGFP-CVB at MOI of 0.001, respectively, for 24 h (scale bars = 100 µm). (**D**) Western blots detecting VP1 and IFITM2/3 in HeLas treated with *siIFITM2* for 48 h before infecting with EGFP-CVB at MOI of 0.001, respectively, for 24 h. Pon S stain is shown below. Densitometric quantification of VP1 and IFITM2/3 is shown to the right. (**E**) Western blots detecting VP1 and IFITM3 in HeLas treated with *siIFITM3* for 48 h before infecting with EGFP-CVB at MOI of 0.001, respectively, for 24 h. Pon S stain is shown below. Densitometric quantification of VP1 and IFITM2/3 is shown to the right. (**F**) Viral plaque assays enumerating infectious viral titers in tissue culture media from cells in C. (Student’s t-test (**D**,**E**), ANOVA (**A**,**B**,**D**–**F**); n = 3 per group; * *p* < 0.05, ** *p* < 0.01, *** *p* < 0.001, **** *p* < 0.0001. Error bars represent standard deviation.)

Given that DEHP exposure may perturb virally mediated interferon responses, we next determined whether interferon-stimulated genes (ISGs) like interferon-induced transmembrane proteins (IFITMs) were affected by DEHP. IFITMs are a family of ISGs, and their expression is classically known to be induced by type I interferons [37]. Both IFITM2 and 3 are generally thought to broadly inhibit viral infection; however, their relationship with CVB is poorly understood [38]. We performed Western blots to assess IFITM2/3 protein levels in HeLas and iBECs following DEHP exposure and were surprised to find that they were increased with DEHP whether or not CVB was present (Figure 4A,B). To better understand how these IFITMs might impact CVB infection, we transfected HeLa cells with siRNA targeting either *IFITM2* or *IFITM3* prior to infecting with CVB. Silencing either of these genes substantially reduced infection, as was indicated by decreased viral EGFP and intracellular VP1 levels (Figure 4C–E). Extracellular viral titers were also significantly lower with *siIFITM2* or *siIFITM3* compared with the control group (Figure 4F). These findings suggest that IFITM2 and 3 may actually support CVB infection, and that increased levels of these proteins may contribute to DEHP’s proviral effects.

### 3.5. DEHP Does Not Impair CVB Entry or Subsequent Rounds of Infection

**Figure 5:**
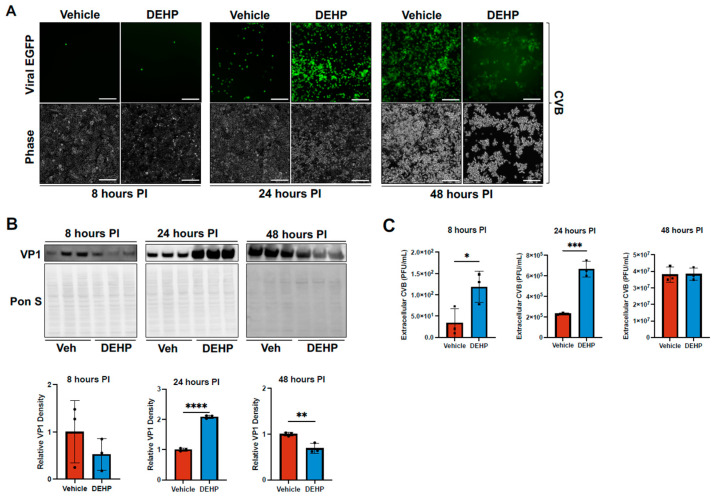
DEHP does not impair CVB entry or subsequent rounds of infection. (**A**) Fluorescence microscopy detecting EGFP expression in HeLa cells treated with 40 μg/mL DEHP or equivalent volume vehicle for 24 h prior to infecting with EGFP-CVB at an MOI of 0.001 for 8 (left), 24 (middle), and 48 (right) hours (scale bars = 200 μm). Corresponding phase contrast images are shown below. (**B**) Western blots detecting viral capsid protein VP1 in lysates from cells in (**A**) (Western blot image exposure and contrast were separately adjusted for each time point). Ponceau S (Pon S) stain is shown below. The densitometric quantifications of VP1 are shown below. (**C**) Viral plaque assays enumerating infectious viral titers in tissue culture media from cells in (**A**). (Student’s t-test; n = 3 per group; * *p* < 0.05, ** *p* < 0.01, *** *p* < 0.001, **** *p* < 0.0001. Error bars represent standard deviation.)

IFITM2 and 3 have previously been implicated in inhibiting the entry of certain enveloped viruses, including hepatitis C virus, influenza A, and SARS-CoV-2 [39,40,41]. To determine whether extracellular viral accumulation of CVB was potentially a result of impaired entry, we treated HeLa cells with DEHP prior to infecting with EGFP-CVB at MOI 0.001 as we have in previously experiments; however, we assessed infection at 8, 24, and 48 h post-infection. We found that, at 8 h post-infection, the viral EGFP signal was minimal and undiscernible between infected vehicle-treated and infected DEHP-treated groups (Figure 5A). Consistent with this, cellular VP1 levels were not significantly different (Figure 5B); however, extracellular viral titers were significantly increased with DEHP (Figure 5C). At 24 h post-infection, as we saw previously, the viral EGFP expression was dramatically higher in the DEHP group, as were cellular VP1 levels and extracellular viral titers (Figure 5A–C). At 48 h post-infection, whereas infected vehicle-treated cells were still mostly viable and displayed a progressive increase in viral EGFP (Figure 5A), the infected DEHP-treated cells were almost completely dead and showed a decay in viral EGFP due to a lack of host cell viability. Cellular VP1 was modestly reduced in the DEHP group and extracellular viral titers were not significantly different between infected vehicle-treated and infected DEHP-treated groups, likely due to the drop off in live cells in the latter group (Figure 5B,C). To rule out that potential DEHP-mediated cytotoxicity was killing the cells at 48 h of infection, we also treated mock-infected cells with and without DEHP for 72 h and saw no observable difference in cell density or morphology (Supplemental Figure S3). This infection time course demonstrates that DEHP-treated cells indeed are still susceptible to subsequent rounds of infection, thus viral entry appears to be intact despite DEHP-mediated increases in IFITM2 and 3.

### 3.6. DEHP Has No Effect on Murine Cytomegalovirus Replication but Increases Group B Streptococcal Attachment and Invasion

**Figure 6:**
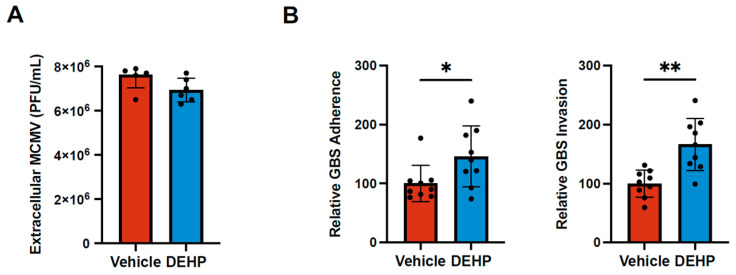
DEHP has no effect on murine cytomegalovirus replication but increases group B streptococcal attachment and invasion (**A**) Plaque assays enumerating infectious viral titers in tissue culture media from 10.1 mouse embryonic fibroblasts treated with vehicle or DEHP prior to infecting with MCMV at an MOI of 0.001. Supernatants were harvested and titered once 50% cytopathic effect was detected. (**B**) GBS adherence and invasion measurements on iBECs treated with vehicle or DEHP before infecting with GBS at an MOI of 10. Adherence and invasion were measured at 30 min and 2 h PI, respectively. (Student’s t-test; n = 6 or 9 per group; * *p* < 0.05, ** *p* < 0.01. Error bars represent standard deviation.)

To determine whether DEHP could alter infection with other unrelated pathogens, we tested effects of DEHP on infection with the DNA virus, murine cytomegalovirus (MCMV), and the bacterium group B streptococcal (GBS). These pathogens were selected because they are very commonly used to study associated human disease, and our group has extensive collective experience in handling them. Additionally, their dissimilarity to CVB allows us to obtain a general view of how broadly DEHP can influence infection. MCMV is a double-stranded DNA virus that establishes latency in mice and is used as a model of human CMV infection and spread [42,43,44,45]. Treating cells with 40 µg/mL DEHP did not impact MCMV replication in infected 10.1 mouse embryonic fibroblasts (Figure 6A). Additionally, we separately tested the effects of DEHP on GBS infection of iBECs. GBS is a common Gram-positive bacterium that colonizes the genitourinary and gastrointestinal tracts of 10–30% of pregnant women [46]. Peripartum infections are a major risk factor for potentially fatal neonatal meningitis unless antibiotics are appropriately administered [47]. Some parallels can be drawn between GBS infections and CVB infections, as severe illness is heavily skewed towards very young children, and both are frequently associated with meningitis [47,48,49,50,51]. As such, both CVB and GBS have developed strategies to penetrate the brain endothelium to gain access to the central nervous system [32,33,52,53]. We had previously demonstrated that iBECs are susceptible to GBS infection, which results in significant disruption of barrier properties [30,31]. iBECs treated with 40 µg/mL DEHP prior to infecting with GBS significantly increased bacterial adherence and invasion (Figure 6B). These data suggest that DEHP can bolster some viral and bacterial infections; however, the effects may be pathogen specific.

## 4. Discussion

Recently, growing concerns have emerged regarding the accumulation of plasticizers and microplastics in the environment and the human health impacts they may have. DEHP is the most widely used member of the phthalate family of plasticizers, with numerous studies identifying the chemical as an endocrine disruptor, probable carcinogen, and immunotoxicant [54,55,56,57,58]. Thus, DEHP exposure is associated with an increasing array of detrimental health manifestations; however, very little is known about the impact DEHP has on infectious disease. In 2021, Lin et al. showed that increased DEHP exposure exacerbated dengue virus infection and disease, and raised the question of whether DEHP could pose a risk for other pathogens [12].

CVB infections commonly occur worldwide and, generally, will result in subclinical illness. However, severe and sometimes fatal diseases such as aseptic meningoencephalitis, myocarditis, and pancreatitis can occasionally occur and are especially profound in very young children under the age of two years [17,18,19]. The risk factors associated with exacerbated CVB infections are poorly understood and generally thought to involve genetic, lifestyle, or environmental factors [20,21]. Elucidating these aspects could provide information as to why certain individuals exhibit higher susceptibility to severe CVB-induced illness.

In this study, we observed that DEHP can profoundly enhance CVB infection in a wide range of cultured cell types, including HeLa cells, iBECs, Caco-2 cells, and HL-1s. In several of these cell types, we observed heightened viral spread as indicated by increased infected cell numbers and disproportionately higher amounts of extracellular virus compared with intracellular viral protein. Furthermore, our data suggest that DEHP may subvert virally induced interferon signaling, and we hypothesize that this may contribute to exacerbated infection. Interestingly, while DEHP blunted IRF7 induction in response to CVB infection, we found that DEHP increased levels of IFITM2 and 3. These IFITMs have generally been characterized as antiviral, particularly in relation to enveloped viruses wherein membrane fusion events are altered to inhibit viral entry into the cytoplasm [39,59,60]. However, when we silenced IFITM2 or IFITM3, we found CVB infection to be significantly reduced, suggesting that these IFITMs support CVB infection. These particular IFITMs have been shown to have roles in endosomal and autophagosomal function during viral and bacterial infection [39,61,62]. We and others had previously reported that CVB can enter and hijack autophagosomes to facilitate vesicle-based viral egress, thus it is possible that these IFITMs may play a role in promoting these viral processes, especially if membrane fusion and trafficking are altered to favor extracellular vesicle release [47,48,49,50].

IFITMs having potentially proviral effects has been reported for other viruses. For example, SARS-CoV-2 Alpha, Beta, Gamma, and Delta variants of concern (VOCs) were reported to be dependent on IFITM2 for replicating in lung cells, as depleting IFITM2 inhibits the production of infectious progeny [63]. Nonetheless, further studies are needed to elucidate the role of these IFITMs during CVB infection, as very little is known about the relationship between CVB and IFITMs. Furthermore, because the DEHP-mediated increase in these IFITMs occurs in a setting in which IRF7 levels are reduced, this may suggest that DEHP-elicited IFITM induction is independent of type I interferon signaling. Interferon-independent induction of IFITMs has been previously described. For example, human CMV (HCMV) has been described to be able to induce ISGs (including IFITMs) independent of type I interferon signaling either by directly promoting phosphorylation of IRF3, activating the JAK-STAT pathway, or promoting MAVS signaling [64,65,66,67,68,69]. Moreover, it was shown that overexpressing IFITMs 1, 2, and 3 does not seem to be antiviral to HCMV, but rather may bolster it [70,71]. Another study investigating the RNA virus Sendai virus showed that IFITM3 does not limit infection and actually promotes the autophagic degradation of IRF3, limiting type I interferon responses [13]. Thus, interferon-independent IFITM activation has been described in a number of contexts; however, mechanisms of how DEHP may induce their activation remain unclear.

There are likely numerous aspects that contribute to DEHP’s proviral properties. For example, DEHP has been shown to increase the generation and accumulation of reactive oxygen species (ROS), and it has also been reported that ROS bolsters enteroviral replication [72,73]. On a similar note, it was reported that DEHP can disrupt mitochondrial dynamics and function, which not only could contribute to oxidative stress, but also impair mitochondria-resident antiviral machinery such as the nucleotide-binding domain, leucine-rich repeat-containing protein 3 (NLRP3) inflammasome, and mitochondrial antiviral signaling (MAVS) protein [74,75]. Furthermore, as an extension of DEHP-mediated mitochondrial disruption, mitophagy has been demonstrated to also be amplified, and we had previously reported that mitophagy is a crucial component of CVB egress via extracellular vesicles [76,77].

Another surprising finding in this study was the observation that DEHP’s effects appear to extend beyond just being proviral, as we also observed significantly increased adherence and invasion with GBS when DEHP is present. It is not clear how DEHP promotes CVB infection and GBS attachment and invasion. It will be important to elucidate mechanisms contributing to enhanced viral egress and suppressed cellular antiviral responses. Our data indicated that DEHP had no effect on MCMV infection, which suggests that DEHP is not broadly proviral. Given that DEHP also promotes dengue virus infection (another RNA virus), it would be interesting to determine what effects DEHP has on RNA virus-specific immunity such as double-strand RNA surveillance. It should be noted that MCMV studies were performed in a mouse embryonic fibroblast line, thus we cannot rule-out the possibility that the cell type used may have influenced the lack of observed anti-MCMV effects, as we did not comparatively evaluate CVB infection in those cells. Additionally, it is unknown whether a common pathway underlies the observed increases in both CVB and GBS infection following DEHP treatment, or if unrelated antiviral and antibacterial systems are impacted separately. In all, there exist few studies exploring the impact that DEHP has on infectious disease. For this current study we selected MCMV and GBS as candidate DNA viral and bacterial pathogens to determine whether DEHP might confer similar infection-bolstering effects. We chose these, in part, because this would offer us a better understanding of the effects that DEHP has on a broader range of human pathogens. However, knowing the proviral effects that DEHP has on CVB infection, it would be important for future studies to investigate effects on other important picornaviruses such as rhinoviruses EV-D68 and EV-71. We surmise that, due to similarities in their virology, DEHP could also enhance these infections; however, that is beyond the scope of this present study, which focuses on CVB. Further interrogating cellular alterations that arise from DEHP exposure would be valuable in identifying whether this substance is a possible environmental risk factor for not only CVB infection but potentially for a larger range of viral and bacterial pathogens. Additionally, elucidating these DEHP-mediated mechanisms will no doubt help inform novel strategies to treat CVB-mediated diseases.

## Supplementary Materials

The following supporting information can be downloaded at: https://www.mdpi.com/article/10.3390/v16121821/s1.
