# Supplementary figures and images for: Common Chemical Plasticizer Di(2-Ethhylhexyl) Phthalate Exposure Exacerbates Coxsackievirus B3 Infection
Source: Viruses. 2024 Nov 23;16(12):1821. doi: 10.3390/v16121821 (PMC11680387; doi:10.3390/v16121821)

**A**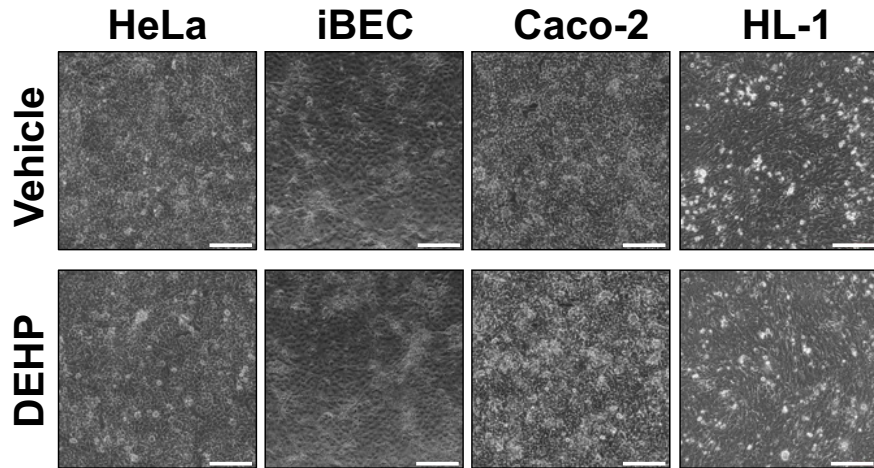**B**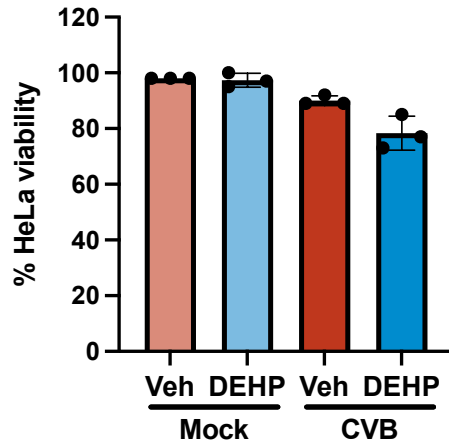

Supplement: Supplementary file 1 [file viruses-16-01821-s001.zip › Supp figure 1.pdf]

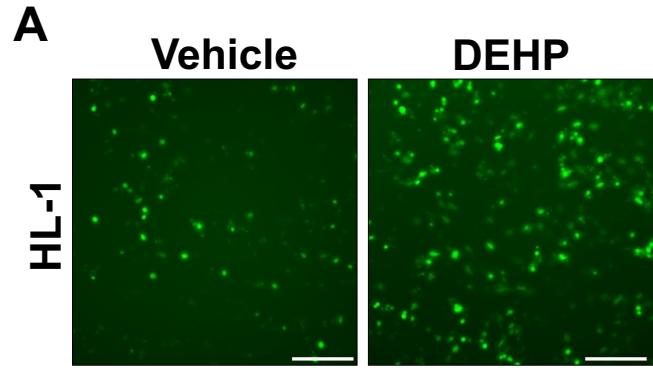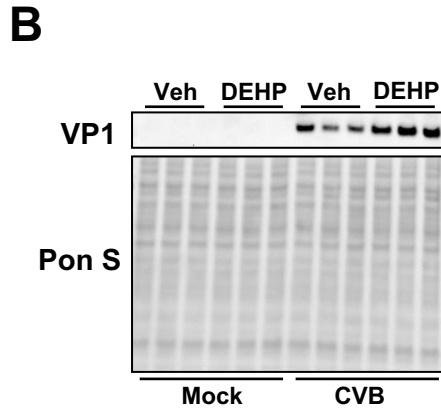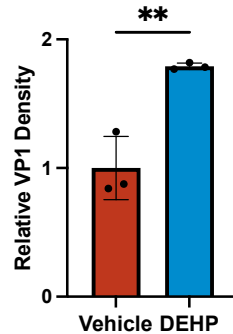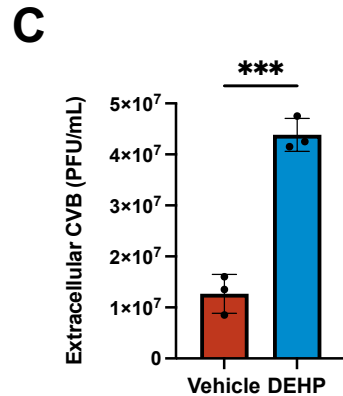

Supplement: Supplementary file 1 [file viruses-16-01821-s001.zip › Supp figure 2.pdf]

**72 hours treatment**

**Vehicle**

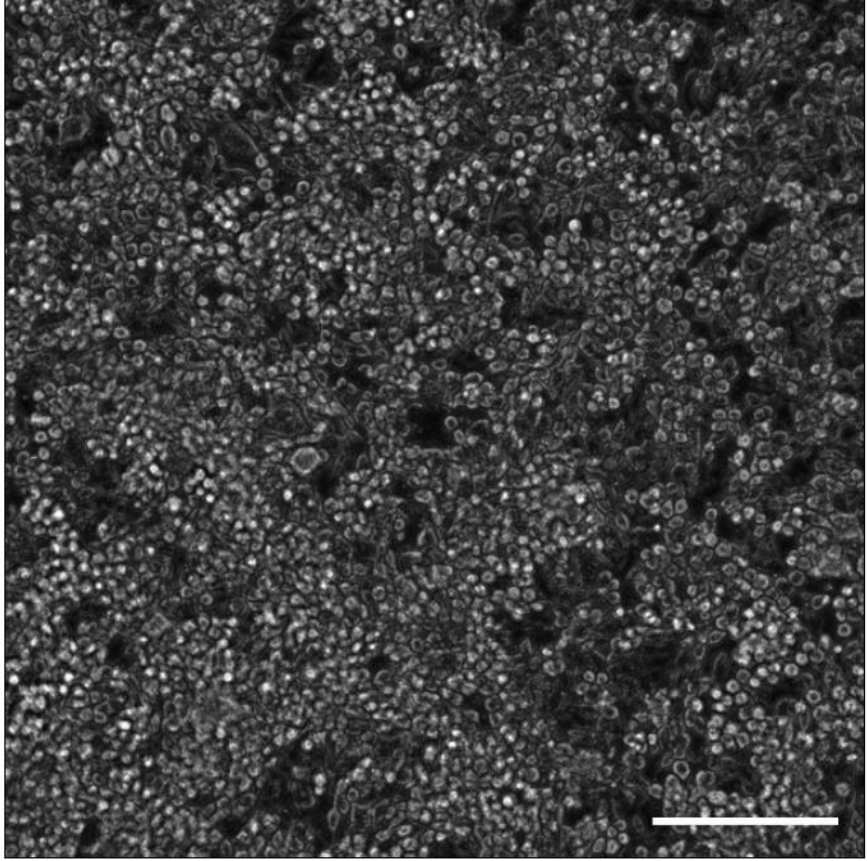

**DEHP**

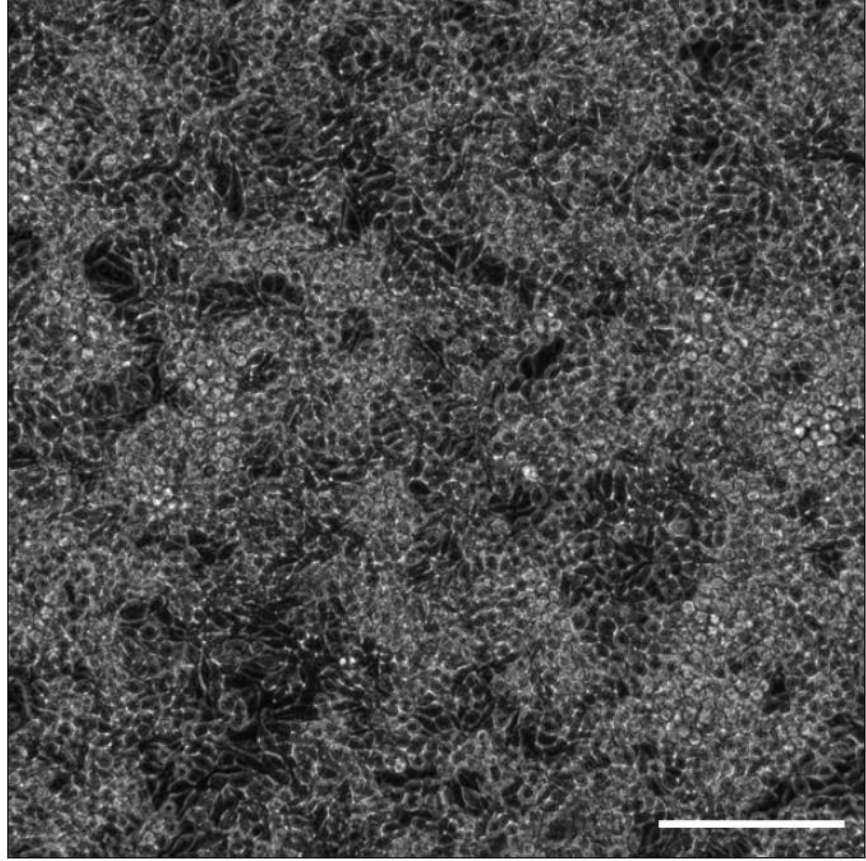

**Mock**

Supplement: Supplementary file 1 [file viruses-16-01821-s001.zip › Supp figure 3.pdf]
